# Thromboelastometry-Based Profiling of Haemostatic Alterations in Neonatal Sepsis by Causative Pathogens

**DOI:** 10.3390/antibiotics14010101

**Published:** 2025-01-17

**Authors:** Rozeta Sokou, Eleni A. Gounari, Konstantina A. Tsante, Aikaterini Konstantinidi, Maria Lampridou, Martha Theodoraki, Anastasios G. Kriebardis, Sotirios P. Fortis, Nicoletta Iacovidou, Andreas G. Tsantes

**Affiliations:** 1 Neonatal Intensive Care Unit, “Agios Panteleimon” General Hospital of Nikea, 18454 Piraeus, Greece; akonstanti@med.uoa.gr (A.K.); marla@med.uoa.gr (M.L.); thmartha@med.uoa.gr (M.T.); 2 Neonatal Department, Aretaieio Hospital, National and Kapodistrian University of Athens, 11528 Athens, Greece; niakoid@med.uoa.gr; 3 East Sussex Hospitals NHS Trust, Hastings TN37 7PT, UK; eleni.gounari@nhs.net; 4 Department of Biomedical Sciences, University of West Attica, 12243 Athens, Greece; dml23026@uniwa.gr (K.A.T.); akrieb@uniwa.gr (A.G.K.); sfortis@uniwa.gr (S.P.F.); 5 Laboratory of Haematology and Blood Bank Unit, “Attikon” Hospital, National and Kapodistrian University of Athens Medical School, 12462 Athens, Greece; andreas.tsantes@yahoo.com; 6 Microbiology Department, “Saint Savvas” Oncology Hospital, 11522 Athens, Greece

**Keywords:** neonatal sepsis, Gram-positive bacteria, Gram-negative bacteria, neonatal haemostasis, thromboelastometry

## Abstract

**Background**: Neonatal sepsis is a serious condition with high mortality, especially in premature and low-birth-weight neonates. This study aims to examine whether the haemostatic profile of neonates with sepsis defers depending on the type of bacteria (Gram-positive or Gram-negative), by using the method of Rotational Thromboelastometry (ROTEM). **Methods**: This single-centre prospective cohort study was conducted on 128 neonates with sepsis, including 95 cases caused by Gram-negative pathogens and 33 cases caused by Gram-positive bacteria. All participants were hospitalised in the Neonatal Intensive Care Unit (NICU). ROTEM parameters were compared between neonates with Gram-positive and Gram-negative infections. **Results**: The ROTEM parameters were found to be significantly different between neonates suffering from Gram-positive versus Gram-negative infections, with Gram-positive pathogens associated with an increased clotting potential compared to Gram-negative pathogens. This is reflected in the higher ROTEM values such as A10, α-angle, and MCF in the EXTEM and INTEM assays. Multivariant analysis showed that Gram-positive infections were linked to increased clot thickness at 10 min (coefficient: 8.9, CI: 2.8–15.0, *p* = 0.004), higher maximum clot stability (coefficient: 10.4, CI: 4.3–16.6, *p* = 0.001), and a bigger α-angle (coefficient: 8.0, CI: 2.7–13.2, *p* = 0.003). Similar findings were observed in the INTEM assay parameters. **Conclusions**: Neonatal sepsis caused by Gram-positive bacteria leads to a hypercoagulable haemostatic state, whereas neonates with sepsis caused by Gram-negative bacteria exhibit a more hypocoagulable profile and a higher incidence of haemorrhagic episodes. These findings provide valuable insights into the haemostatic disorders associated with sepsis, and may aid in developing an individualised approach for the treatment of those disorders, dependent on and adapted for the specific type of causative organism.

## 1. Introduction

Sepsis is a life-threatening condition caused by a dysregulated host response to an infection and characterised by severe multi-organ dysfunction [1]. Despite neonates being particularly susceptible to sepsis, incidence data for this age group are limited or lacking in many countries [2]. According to the 2016–2017 Global Burden of Disease (GBD) Study report, it is estimated that there are annually 1.3 million cases of neonatal sepsis recorded globally (95% CI 0.8–2.3 millions) [3], resulting in approximately 203,000 sepsis-related deaths (95% CI 178,700–267,100) [4]. In 2018, it was estimated that 375,000 neonatal deaths were attributed to sepsis, a number representing 15% of all neonatal deaths globally [5]. The prognosis of neonatal sepsis is largely dependent on timely recognition and appropriate therapeutic management, but the clinical signs and symptoms of sepsis are often non-specific, making diagnosis challenging and potentially delaying treatment [6,7]. Early recognition is vital, as delayed or inappropriate use of antibiotics can severely impact the morbidity and mortality outcomes of sick neonates with sepsis. On the other hand, the overuse of antibiotics can adversely affect the microbiome, contribute to organ dysfunction, or cause idiosyncratic toxicities [8,9]. The variations in host responses and the virulence mechanisms of different pathogens must be considered when treating patients with bacteraemia. The attributes of the causative pathogen hold prognostic significance. Studies reported higher mortality rates for infections caused by Gram-negative bacteria (10–40%) compared to those caused by Gram-positive bacteria (7–27%) [10,11]. Moreover, antimicrobial resistance (AMR) has risen significantly worldwide over the past decade. Currently, approximately 50–70% of Gram-negative bacteria responsible for neonatal sepsis are multidrug resistant (MDR), leading to greater morbidity and mortality, extended hospital stays, and increased healthcare costs [12]. Additionally, there is an urgent need for the development of new therapeutic strategies beyond conventional antibiotics, as the prompt initiation of appropriate antimicrobial therapy is vital for the effective management of sepsis [13]. A targeted therapy with an individualised dosing scheme and duration of treatment, guided by diagnostic biomarkers and patient responses to treatment, could help rationalise antibiotic use without increasing risks of undertreatment, and therefore, could contribute to increased effectiveness and safety. Validation of such biomarkers in different geographical and economic environments is integral to ensure that their sensitivity and specificity remain reliable in different clinical conditions and aetiologies of sepsis. The development of such diagnostic tests would aid appropriate escalation or de-escalation of treatment in patients with suspected sepsis. Blood culture remains the gold standard in the diagnosis of sepsis [14,15]. Nevertheless, its limitations are the increased timeframe to obtain results, which ranges from 48 to 72 h or more, and false negative results, especially from neonates with low birth weight, where obtaining adequate blood volume for culture may be difficult. Diagnosis remains challenging in the early stages of disease due to the non-specific nature of clinical signs and symptoms, and therefore, relies largely on laboratory tests such as full blood count (FBC) and serum levels of C-reactive protein (CRP), rather than the time-consuming blood culture [16,17]. Recent studies have highlighted the role of procalcitonin (PCT) [18,19] and persepsin as potential biomarkers with high sensitivity and specificity for the diagnosis of neonatal sepsis [20,21,22,23,24]. However, it remains unclear how laboratory markers differ according to the onset of infection and the type of bacteria, either Gram-positive or Gram-negative. A recent study found that neonatal late-onset sepsis (LOS) caused by Gram-negative bacteria is associated with higher CRP levels and lower platelet (PLT) numbers, with the authors suggesting that both CRP and PLT values can be useful biomarkers for differentiating sepsis caused by Gram-negative versus Gram-positive bacteria in term neonates [25].

The activation of clotting pathways is frequently observed in severely unwell patients with sepsis. Sepsis disrupts the physiological fine balance between prothrombotic and antithrombotic factors, severely disturbing the homeostasis of the coagulation system and leading to the increased production of thrombin, reduced anticoagulant activity, and suppression of fibrinolysis. This condition is known as Sepsis-Induced Coagulopathy (SIC) and it is a frequent and severe complication observed in 24% of patients with septicaemia and 66% of patients with septic shock. The development of SIC is linked with adverse clinical outcomes and increased mortality, as it leads to disseminated intravascular coagulation (DIC), multi-organ dysfunction syndrome (MODS), and increased clotting risk [26,27,28]. The close relationship between haemostatic disorders and host inflammatory responses constitutes an important link in the pathogenesis of severe infection and sepsis [29]. The clotting activation pathways, along with the regulatory mechanisms developed by bacteria in response, could be the targets of novel therapeutic strategies for the treatment of infectious diseases. Despite this, there are only few studies that have assessed the haemostatic profile of septic patients depending on the type of pathogen causing sepsis, and these are virtually non-existent in the neonatal population [30,31]. The aim of this study is to evaluate whether the haemostatic profile of septic neonates is affected by the type of bacteria causing sepsis. More specifically, this study intends to find any differences between the values of rotational thromboelastometry (ROTEM) parameters in septic neonates with either Gram-positive or Gram-negative sepsis and assess the potential of ROTEM values in differentiating Gram-positive from Gram-negative bacterial infections.

## 2. Results

### 2.1. Baseline Characteristics of Study Cohort

Overall, 128 neonates with septicaemia were included in the study. Among them, 95 (74.2%) had an infection caused by Gram-negative pathogens, while for 33 (15.8%) neonates, the causative pathogen was Gram-positive bacteria. The most common pathogens in neonates with Gram-negative infections included *Klebsiella* spp. other than *Klebsiella pneumioniae* (*n* = 28. 29.4%), Enterobacter cloacae (*n* = 27, 28.4%), *Klebsiella oxytoca* (*n* = 18. 18.9%), and *Pseudomonas aeruginosa* (*n* = 6, 6.3%). The most common pathogen in neonates with Gram-positive infections was *Staphylococcus epidermidis* (*n* = 22, 66.6%), followed by *Staphylococcus haemolyticus* (*n* = 6, 18.1%). The median gestational age was similar for both groups (medians: 32.0 weeks, *p* = 0.91), while neonates with Gram-positive and Gram-negative pathogens also did not differ in terms of sex (males: 63.6% vs. 60%, *p* = 0.92) or birth weight (medians: 1490 g vs. 1480 g, respectively, *p* = 0.97; Table 1).

Regarding other comorbidities, the incidence of bronchopulmonary dysplasia was similar in neonates with Gram-positive and Gram-negative pathogens (42.1% vs. 45.4%, *p* = 0.50), as well as the incidence of respiratory distress syndrome (63.6% vs. 80.0%, *p* = 0.059), and intraventricular haemorrhage (44.2% vs. 51.4%, *p* = 0.30). However, acute kidney injury was less common in neonates with Gram-positive bacteria than in those with Gram-negative bacteria (15.1% vs. 34.7%, *p* = 0.034; Table 1).

Regarding the laboratory findings in the two study groups, neonates that had a Gram-positive infection had a higher platelet count (median: 169.0 × 10^3^/mL vs. 60.0 × 10^3^/mL, *p* = 0.005), lower white blood cell count (median: 9.6 × 10^3^/mL vs. 13.4 × 10^3^/mL, *p* = 0.037), and lower CRP levels (median: 30.5 mg/L vs. 53.9 mg/L, *p* = 0.005) compared to neonates with an isolated Gram-negative pathogen. Moreover, neonates with Gram-positive pathogens had a lower TOLLNER score (median: 7.0 vs. 11.5, *p* < 0.001), lower nSOFA score (median: 2.0 vs. 5.0, *p* = 0.001), and lower modified NEOMOD score (median: 5.0 vs. 8.9, *p* = 0.001) compared to neonates with Gram-negative bacteria (Table 2).

These findings indicate that Gram-negative bacteria were associated with a worse clinical presentation and more severe infections. This is further supported by the fact that the mortality rate was higher in neonates with Gram-negative pathogens (16.6% vs. 3.0%, *p* = 0.044; Table 1).

### 2.2. ROTEM Parameters

**Figure 1:**
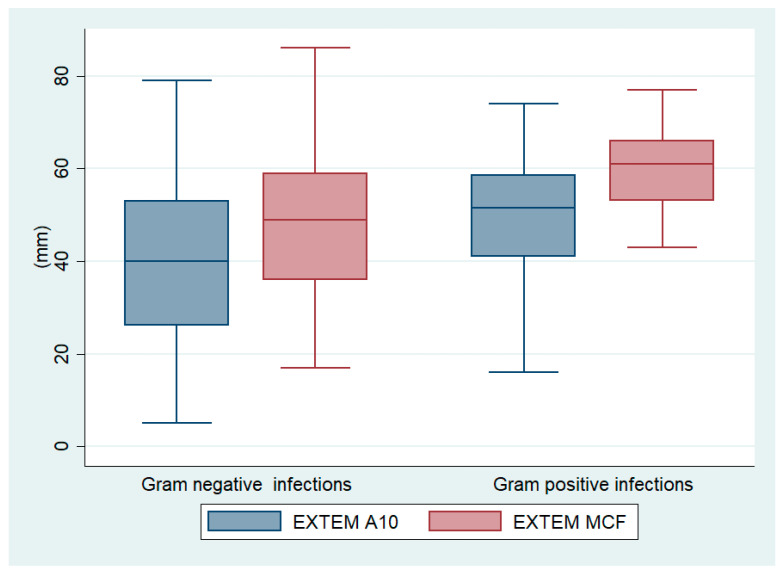
Boxplots of EXTEM clot amplitude at 10 min (A10) and maximum clot firmness (MCF) in neonates with Gram-positive and Gram-negative infection.

Several ROTEM parameters deferred between neonates with Gram-positive and Gram-negative infections, indicating that Gram-positive pathogens may be associated with an accelerated coagulation potential compared to Gram-negative pathogens (Table 3). Specifically, neonates with Gram-positive infections had higher clot amplitude at 10 min (EXTEM A10 medians: 51.5 mm vs. 40 mm, *p* = 0.001), higher maximum clot firmness (EXTEM MCF medians: 61 mm vs. 49 mm, *p* < 0.001), and higher alpha angle (EXTEM alpha°: 76.0° vs. 72.5°, *p* = 0.004) compared to neonates with Gram-negative infections (Figure 1). Moreover, the same hypercoagulable status was evident from the results of the INTEM and FIBTEM methods of ROTEM analysis, since neonates with Gram-positive infections had higher INTEM A10, INTEM MCF, and FIBTEM A10, and a higher alpha angle in INTEM and FIBTEM assays.

The strong association between Gram-positive pathogens and a higher coagulation dynamic, as reflected by a higher clot amplitude at 10 min and a higher maximum clot firmness, was also supported by the results of the multivariable linear regression analysis (Table 4).

Specifically, the multivariable regression analysis adjusted for sex and birth weight revealed that an infection by Gram-positive pathogens was associated with a higher EXTEM clot amplitude at 10 min (coefficient: 8.9, 95% Confidence Interval [CI]: 2.8–15.0, *p* = 0.004), a higher EXTEM maximum clot firmness (coefficient: 10.4, 95% Confidence Interval [CI]: 4.3–16.6, *p* = 0.001), and a higher EXTEM alpha angle (coefficient: 8.0, 95% Confidence Interval [CI]: 2.7–13.2, *p* = 0.003). The same INTEM parameters were associated with Gram-positive pathogens, as shown in Table 4.

## 3. Discussion

In this study, and for the first time in the international literature, there was an evaluation of the haemostatic profile of septic neonates using the ROTEM method. The analysis was performed based on the type of pathogen, differentiating between Gram-negative and Gram-positive bacteria. ROTEM parameter assessment of neonates with both Gram-positive and Gram-negative septicaemia revealed a significantly hyper-coagulant state in neonates with Gram-positive sepsis compared to neonates with Gram-negative pathogens, which were characterised by a more hypo-coagulant profile and more episodes of haemorrhage. This approach enables a more individualised understanding of haemostatic dysfunction related to sepsis and provides valuable information for targeted therapeutic interventions depending on the aetiology of bacterial sepsis.

Sepsis constitutes one the main causes of morbidity and mortality in children worldwide [1,32]. The mechanisms via which bacteria cause sepsis and septic shock include both a bacterial component (such as cell wall and bacterial secretions) and the host’s immune and tissue level responses [33,34]. The severity of sepsis caused by Gram-positive versus Gram-negative bacteria has been debated [13,35]. A recent systematic review and meta-analysis showed that sepsis caused by Gram-negative bacteria is linked with higher serum concentrations of inflammatory markers and greater disease severity compared to Gram-positive sepsis [36]. Both animal studies and clinical models have shown that the haemodynamic response to sepsis is similar in both those two types of bacteria [37,38,39]. This observation led to the hypothesis that the proinflammatory mediators that participate in the pathophysiology of sepsis are identical, irrespective of the type of causative bacteria, and consequently led to the expectation that the inhibition of certain mediators may have a similar effect on patients with both Gram-positive and Gram-negative sepsis. Despite that, recent studies have shown that this approach is not justified. Specifically, anti-inflammatory factors such as the soluble TNF p75 receptor have been proven effective for patients suffering from Gram-negative sepsis, but can be harmful for patients with Gram-positive sepsis [40,41]. Previous clinical studies that involved administration of high dose corticosteroids, platelet activation factor receptor antagonists, and anti-endotoxin therapy had shown promise in patients with sepsis due to Gram-negative organisms, but no significant effect on infections with Gram-positive bacteria [42]. This differentiation further highlights the significance of a tailored approach to sepsis, one that will take into consideration the type of infectious agent in order to achieve optimal therapeutic results [43].

In the current study, it was found that in neonates, sepsis caused by Gram-positive bacteria causes a hypercoagulable haemostatic response that is characterised by increased values of the ROTEM A10, α-angle, and MCF in the EXTEM and INTEM tests, findings that remained consistent after adjusting for confounding factors such as birth weight, sex, and gestational age, which have previously been shown to affect the haemostatic profile of neonates [44,45,46,47,48,49,50]. These findings indicate an enhanced clot formation in the initial stages of sepsis caused by Gram-positive bacteria in comparison to Gram-negative bacteria, and show that a hypercoagulable state is the initial haemostatic response to sepsis caused by Gram-positive bacteria. Similar findings have been observed in septic premature and adult pigs when infected with Gram-positive bacteria [51,52]. In the case of Gram-negative septicaemia, according to the study findings, neonates have a hypocoagulable profile, reflected in the ROTEM parameters. It should be noted that the ROTEM parameters A10 and MCF integrate the contribution of platelet numbers and functionality, fibrinogen concentration, fibrin polymerization, and factor XIII effect on clot stability [53,54].

In terms of platelets, this study showed that the number of platelets was significantly reduced in neonates suffering from Gram-negative sepsis. Platelets, in addition to their main function in haemostasis, actively participate in host defences by phagocytosis of pathogens and the production of cytotoxic free radicals and oxidative molecules. It is hypothesised that a combination of factors such as diffuse endothelial damage, bacterial or fungal toxins, increased platelet activation, and DIC leads to the increased consumption of platelets during sepsis. The data suggest that a combination of increased platelet destruction with an insufficient increase in platelet production leads to thrombopenia during episodes of sepsis in neonates. Studies have shown that neonates with very low birth weight have a limited capacity to respond to thrombopenia, both in terms of platelet production and thrombopoietin secretion [55]. This inadequate response may be further exacerbated during episodes of sepsis, where the host may also be experiencing reduced energy reserves or liver dysfunction, further compromising their ability to replenish their platelets [56]. Some pathogenic organisms appear to have developed mechanisms that inhibit platelet activation, which helps with their survival inside their host [57]. Many studies have examined thrombopenia in relation to different microorganisms and have suggested using low platelet numbers as an early marker of sepsis [58], but so far there is an ongoing debate, as different studies have yielded different results. Some infectious agents such as Gram-negative bacteria, fungi, and coagulase negative staphylococci have been reported to significantly impact platelet numbers when they are causing sepsis [57,59,60,61]. In a study by Eissa and El-Farrash [61], neonates with Gram-negative sepsis had the lowest numbers of platelets and reticulated platelets (RP), and highest levels of thrombopoietin (TPO) and reticulated platelet percentage (RP%), followed by those with fungal sepsis. The number of platelets was inversely correlated with TPO levels and RP% and was directly associated with absolute RP numbers. Scheifele et al. [62] examined the association between endotoxinaemia and thrombopenia during episodes of necrotising enterocolitis (NEC). In their study, 49% of the patients had detectable endotoxin-like activity (ELA) and 28% had a platelet nadir below 100.000/mm^3^. Of those with detectable ELA, 47% developed thrombopenia, whilst only 9.5% of patients without detectable ELA developed thrombopenia. In another study, Rowe et al. [63] examined 93 post-op paediatric surgical patients and noted that 71% of the patients with Gram-negative sepsis had platelet numbers below 100.000/mm^3^, whereas all non-septic patients, or those with Gram-positive sepsis, had platelet numbers above 150.000/mm^3^. It was additionally shown that platelet numbers recovered when patients received appropriate treatment for sepsis.

The thrombopenia observed in Gram-negative sepsis may be due to increased platelet destruction caused by mechanisms such as binding and antibody-mediated activation. Lipopolysaccharides (LPS), which constitute the main ingredient of Gram-negative bacteria cell walls, have been proven to cause thrombopenia in experimental models in acellular extracts. The inflammatory response caused by LPS may activate the consumption and destruction of platelets, aggravating the haemostatic instability in Gram-negative sepsis [64,65,66,67]. Platelets express many receptors on their surface that bacteria can potentially bind to, including complement receptors, FcγRII, Toll type receptors (TLR), and integrins such as GPIIb–IIIa or GPIb that are typically part of the haemostatic response. Bacteria bind to these receptors either directly or indirectly, through fibrinogen, fibronectin, complement protein C1q, von Willebrand factor, etc. A few studies have noted the ability of activated platelets to internalise bacteria such as *Staphylococcus aureus* or *Porphyromonas gingivalis* [68]. Recently, the increased expression of GPIIb/IIIa surface receptors on activated platelets has been recognised in infections from staphylococcus aureus, signifying direct and indirect platelet activation as part of an «immunothrombosis» response to bacterial invasion [69,70,71,72]. During systematic inflammation, P-selectin is expressed on platelet surfaces, facilitating platelet aggregation and the binding of platelets to leucocytes, along with tissue factor expression by monocytes, both contributing to the initial platelet activation through the formation of Neutrophil Extracellular Traps (NETs), which trap bacteria, concentrate antibacterial agents, and enhance thrombosis [70]. Additionally, the fact that bacteria can bind to platelets through receptors that participate in the haemostasis cascade indicates that they can cause platelet aggregation, something already described for *Streptococcus sanguinis*, *S. epidermidis*, and *C. pneumoniae* [68,73].

The best-defined antibacterial function of haemostasis is fibrin formation at the site of infection, which immobilises bacteria and prevents their spread to adjacent tissues. It has been known for decades that the fibrin network restricts the spread of bacteria to the circulatory system and reduces the risk of systemic infections [74]. Mice that have fibrinogen deficiency were observed to have increased susceptibility to infections from *streptococcus pyogenes* (group A streptococci [GAS]) due to reduced fibrin formation [75]. For pathogens such *as Staphylococcus aureus* and *Escherichia coli*, their entrapment in the fibrin clot is dependent of factor XIII (FXIII), as these bacteria are directly linked to the fibrin fibres through anchor points that are cross-linked with factor XIII. In factor XIII, deficiency bacteria are more loosely held within the fibrin network [76]. Additionally, FXIII contributes to *GAS* entrapment in the site of infection, thus activating an eradication mechanism through the production of antimicrobial peptides [77]. *Staphylococcus aureus* appears to be able to recruit and activate FXIII within fibrin clots that are formed between the von Willebrand factor-binding protein (vWbp) and coagulase (Coa). The activated FXIII stabilises fibrin fibres, rendering them resistant to degradation and enhancing thrombi formed within abscesses, thus potentially protecting the bacteria from the host’s immune responses [78]. Fibronectin, which is a FXIIIa substrate, is linked with these clots, improving the mechanical qualities of fibrin [79,80]. In addition, Fibronectin-binding protein A (FnbpA) acts as a substrate to FXIIIa, resulting in the homopolar connection of FnbpA with fibronectin and fibrin/fibrinogen [81]. This process not only stabilises fibrin clots, preventing destruction from phagocytes, but also helps *S. aureus* to embed its own proteins within the fibrin network, allowing for immunological escape and survival of the bacteria within the host’s tissues [82]. These mechanisms point to the significance of FXIII not only for the stabilisation of the clot, but also in the body’s immunological responses. Our results are consistent with the literature that has shown FXIII to play an important role in disorders of haemostasis during sepsis, some of them specific to certain infectious agents.

Initially, bacteraemia and endotoxinaemia lead to increased fibrinolytic activity due to the release of plasminogen activators from endothelial cells. However, this pro-fibrinolytic activity is immediately followed by the suppression of fibrinolysis. It appears that in sepsis, fibrinolysis, though activated, is insufficient to counteract fibrin formation, and that increased fibrin formation can cause organ dysfunction and even death [83,84,85,86]. It is worth noting that in our study, septic neonates due to Gram-positive bacteria exhibited increased fibrinolytic activity compared to those with Gram-negative sepsis, as expressed by the EXTEM LI60 values in the ROTEM test. When we adjusted our analysis for confounding variables, the statistical difference in the LI60 values between the two groups disappeared. This finding may be due to the reduced levels of fibrinolysis inhibitors in premature neonates, which is characteristic of developmental haemostasis. The population of our study was mainly premature neonates with a mean gestational age of 32 weeks (similar for both groups). In preterm neonates, haemostatic system immaturity is expressed through reduced activity of fibrinolysis inhibitors, which can explain the absence of statistically significant LI60 after adjusting for cofounders such as gestational age, birth weight, and sex [44]. Moreover, because viscoelastic tests assess the haemostatic system holistically, the parameters assessing fibrinolysis do not constitute a quantification of fibrinolytic activity as such, but rather express the equilibrium between clot formation and degradation [87]. Thus, a disordered fibrinolytic response may exist, but it is overshadowed by the intensely hypo-coagulant profile exhibited by neonates with Gram-negative sepsis. According to a different study assessing the diagnostic value of ROTEΜ in the early recognition of disorders of fibrinolysis in septic neonates [88], the clinical usefulness of parameters LI60 or ML was limited in relation to differentiating between neonates with confirmed sepsis, suspected sepsis, and healthy neonates, and for assessing the outcome of sepsis.

In our study, it was observed that neonates with sepsis from Gram-negative bacteria had a significantly worse clinical condition compared to neonates with Gram-positive sepsis, as shown by their higher TOLLNER, nSOFA, and modified NEOMOD scores. Additionally, the increased incidence of haemorrhages and higher mortality in neonates with Gram-negative pathogens confirm the severity of those cases, and these findings fully align with the hypo-coagulant profile of those neonates in the ROTEM tests. This hypo-coagulant profile reflects disorders of haemostasis that appear to contribute both to the worse outcomes and increased mortality of the neonatal population with Gram-negative sepsis. Also, from previous studies by our research team it has been shown that the ROTEM CT, CFT, MCF, A10, and A20 values reflect not only the severity of the haemostatic deficiency of premature neonates with evident haemorrhagic tendency, but also their outcomes [88,89,90,91,92,93,94,95], findings that align with those of studies on adults with septicaemia [96,97]. Gram-negative bacteria are frequently implicated in the pathogenesis of sepsis and septic shock, but the exact mechanism of that remains uncertain [98,99]. Variations in bacterial virulence mechanisms lead to distinct host responses, including differences in the activation of signalling pathways and the regulation of host cell apoptosis—either stimulation or suppression [13,100,101]. These differences can significantly affect patient outcomes and should be taken into account when managing bacteraemia. Moreover, there is a pressing need to develop innovative therapeutic approaches beyond conventional antibiotics, as initiating appropriate antimicrobial treatment promptly is essential for effectively addressing sepsis.

The activation of the clotting system during sepsis is a common occurrence, caused by a generalised inflammatory response to bacterial antigens, mediated through the tissue factor pathway [102]. The combination of activated clotting pathways with the disruption of anticoagulant mechanisms and inadequate fibrinolysis can lead to the life threatening complication of Disseminated Intravascular Coagulation—DIC, also an abbreviation for “Death is Coming” [103]. During the transition from compensated to decompensated DIC there is a gradual consumption and depletion of clotting factors, accompanied by the formation of microthrombi [102], which can lead to significant haemorrhage, severe complications, multi-organ deficiency, and death. In septic patients, the early recognition and treatment of DIC can prevent those severe complications and improve clinical outcomes. The well-documented hallmarks of DIC on conventional clotting tests are thrombopenia, prolonged prothrombin time (PT), increased fibrin degradation products (FDPs), and low fibrinogen levels as clotting factors are depleted due to excessive clot formation and increased fibrinolysis. These deranged clotting results are typically evident in the advanced stages of DIC, but are usually absent it the early stage [102], rendering them ineffective for the early detection of DIC, prior to its decompensated state and its major haemostatic implications.

Conventional clotting screening (PT, aPTT) does not include cellular elements (platelets, red blood cells, etc.), only provides limited information on segments of the clotting cascade such as initial platelet-less thrombin formation, and cannot provide any information on fibrinolysis or platelet functionality [104,105,106]. Those tests can also be affected by factors such as proteins interacting with very low-density lipoproteins–C-reactive protein complexes (VLDL-C) or antiphospholipid antibodies, thus not presenting a true reflection of the patient’s haemostatic status [107]. Conversely, ROTEM offers a comprehensive evaluation of both the plasma and the cellular clotting system with whole blood analysis, offering data that reflect with greater accuracy the production of thrombin and the actual clotting process. Platelets play an important role in the primary “explosive” thrombin generation, and the use of specialised thromboelastometry parameters can help analyse clot stability and assess fibrinolysis better than conventional clotting tests that are limited in the initial phase of coagulation [104]. The use of the EXTEM, INTEM, and FIBTEM tests combined with the evaluation of ROTEM parameter values offers a more detailed picture of various components of the coagulation cascade and of the process of fibrin polymerization [108]. Additionally, in contrast to conventional clotting screening, ROTEM can detect the fine variations in the balance between coagulation and fibrinolysis in real time, offering a more realistic assessment of the patient’s true haemostatic status, and thus becoming a critical tool in the early recognition and management of DIC, especially in sepsis, where clotting dysfunction can evolve rapidly and with severe consequences [109]. There are cases mentioned in the literature of patients with septicaemia presenting with hypercoagulability detected through ROTEM, despite conventional screening indicating increased risk of haemorrhage [109,110]. This raises concerns given that according to the current protocols, fresh frozen plasma transfusions and clotting factor administration are common practices in septic patients prior to surgical procedures or central line insertion, potentially increasing risk of complications. Furthermore, this highlights the crucial role of ROTEM for the precise evaluation of hyper- or hypocoagulability and the improved clinical management of critical conditions such as sepsis, allowing for more targeted and safer therapies. It is widely recognised that sepsis is a heterogeneous condition, influenced by factors such as host genetics, patient age, comorbidities, source of infection, causative pathogen, and the organ systems involved. These factors collectively determine the host’s response to sepsis, which can vary according to different sepsis endotypes [111]. Despite this variability, certain therapeutic approaches remain consistent across cases. Currently, efforts are underway to identify distinct clinical subgroups of sepsis patients with treatable features, including a coagulopathic subtype, through the use of advanced molecular techniques [112]. Sepsis-induced coagulopathy, a crucial factor to recognise in the clinical management of neonatal sepsis, is often overlooked compared to other forms of organ dysfunction. Monitoring the haemostatic profile is essential in sepsis management, as disruptions in coagulation can significantly impact the patient’s clinical progression. Impaired haemostasis is linked to an increased risk of thrombosis or bleeding complications, which underscores the importance of assessing haemostatic parameters for the early detection and appropriate treatment of sepsis. Increasing understanding of the basic underlying mechanisms allows us to successfully stabilise individual uncompensated subsystems, such as coagulation, in septic neonates. We must learn to embrace these steps in order to achieve better outcomes, particularly in terms of survival, for vulnerable neonates facing this devastating disease.

In terms of the limitations of this study, this is a single-centre study with a relatively small number of patients, so its results should be interpreted with caution. There was no correlation between ROTEM results and conventional clotting tests, as the latter were not performed in the neonates of the study. The variation in the timing of ROTEM measurements even within the first day of development of sepsis and the absence of serial measurements could also affect our results, as sepsis is a dynamic process. The host’s inflammatory response to an infectious organism usually leads to an initial pro-thrombotic phase in the septic patient, but the continuing consumption of clotting factors due to ongoing clotting eventually leads to haemostatic instability. Thus, the evaluation of clotting disorders in acute sepsis is a complex and time sensitive process that is best conducted using serial measurements. On the other hand, the fact that the data are from a single centre limits the effect of different clinical practices and ensures homogeneity in the assessment and the recognition of early clinical signs and symptoms of sepsis.

## 4. Materials and Methods

This is a single-centre, prospective cohort study conducted over a period of 4 years (March 2020–March 2024), which included preterm and term neonates with sepsis hospitalised at the Neonatal Intensive Care Unit (NICU) of the General Hospital of Nikaia, Piraeus. Ethics approval was granted by the Scientific Council on 26 February 2020, (5/24), with the study’s protocol designed, implemented, and reported according to the Declaration of Helsinki. Informed consent was provided by either the parents or guardians of the participating neonates. The protocol included a diagram with the physical examination findings and the laboratory results for every neonate of the study, filled in by two members of the research team (RS, AK).

### 4.1. Definitions

Sepsis was defined as a clinical condition characterised by the isolation of bacteria in the blood culture. Regarding diagnosing sepsis from coagulase-negative staphylococcus (CoNS), two consecutive positive blood cultures with the same CoNS strain, one positive blood culture and concurrent presence of two clinical signs, or laboratory findings indicative of infection, are required [90,113]. The occurrence of specific clinical symptoms and/or laboratory findings indicative of sepsis, which necessitate the initiation of antibiotic treatment, was assessed [113,114]:

Clinical symptoms and signs: (i) temperature instability, (ii) feeding intolerance, (iii) apnea/need for oxygen therapy or mechanical ventilation, (iv) bradycardia/tachycardia, (v) tissue hypoxia, hypotension, (vi) irritability/lethargy, (vii) pallor, jaundice, and haemorrhagic rash. Risk factors for infection, based on perinatal history, include premature and prolonged rupture of membranes (>18 h), maternal fever during labour, and chorioamnionitis.

Laboratory findings: (i) metabolic acidosis or increased lactic acid (>2 mmol/L), (ii) leukocyte count (<5000 or ≥20,000/mm^3^), (iii) absolute neutrophil count >5400/mm^3^ or <1000/mm^3^, (iv) ratio of immature to total neutrophils (I/T) > 0.27 in full-term neonates and >0.22 in preterm neonates, (v) thrombocytopenia (PLT < 100,000/mm^3^), and (vi) increased CRP levels (>5 mg/L).

### 4.2. Exclusion Criteria

Neonates that had previously received fresh frozen plasma or platelet transfusion, with congenital clotting disorders, with personal or family history of haemorrhagic disorders, or known or suspected major congenital or chromosomal disorders were excluded from the study.

### 4.3. Outcome Measurements

All neonates participating in the study had a detailed family, obstetric, perinatal, and neonatal history, obtained along with their demographic data. Any neonate with suspected infection had the following investigations taken prior to starting antibiotic treatment:

Blood culture—a blood sample was obtained from each neonate under aseptic conditions from a peripheral artery and was inoculated with sterile technique in an aerobic culture bottle (BacT Alert PF, Biomerieux, Lyon, France). The microorganisms were identified through the BacT/ALERT system, their Gram-stain, and their growth patterns on specific growth mediums (agar).

Full blood count, acid–base balance, arterial blood sample for pH, anion gap, lactate levels; biochemical profile- liver function tests, urea and electrolytes, creatinine, glucose, calcium, and CRP- were obtained. Depending on their clinical condition, neonates with clinical suspicion of infection may have had a urine culture, cerebrospinal fluid or other biological fluid cultures, or imaging investigations (such as X-ray or ultrasound) to help identify the focus of infection. In addition, repeat samples were collected for infection markers whenever clinically indicated, and up until the antimicrobial therapy was completed.

### 4.4. Rotational Thromboelastometry (ROTEM) Technique

In neonates with suspected infection, ROTEM examination was performed immediately upon clinical suspicion and along with the indicated infection screening. For the ROTEM measurements, the 4-channel «rotational thromboelastometry» device was used (ROTEM^®^ Whole Blood Haemostasis System Rotation Thromboelastography Tem Innovations GmbH, Munich, Germany). All whole blood samples were examined in the ROTEM^®^ within 30 min from venepuncture. The tests’ thromboelastographic curves were recorded for a period of observation of 90 min. ROTEM was performed in whole blood samples and according to the manufacturer’s specifications. The following ROTEM tests were obtained: EXTEM: assessment of extrinsic coagulation pathway; INTEM: assessment of intrinsic coagulation pathway; and FIBTEM: by addition of a potent platelet inhibitor and complete suppression of platelet contribution to clot formation and stabilisation, it can assess the qualitative and quantitative sufficiency of fibrinogen. All blood samples were carefully examined for fibrin clots, with rejection of any sample with a presence of microthrombi. The following EXTEM variables were measured: clotting time (CT, in s), clot formation time (CFT, in s), the clot size–width at 10 min (A10), α-angle (a^o^), maximum clot firmness (MCF, in mm), and clot lysis index at 60 min (LI60, %). Neonates of the study, in addition to their appropriate infection screening, had their Neonatal Sequential Organ Failure Assessment (nSOFA) score [115,116], TOLLNER score [117], and modified NEOMODS score [118] calculated. Incidences of haemorrhage were categorised by their severity using the modified Neonatal Bleeding Assessment Tool (NeoBAT) [119]. The study population consisted of neonates who had not received any antibiotic therapy for a minimum of 48 h prior to their enrolment.

### 4.5. Statistical Analysis

Descriptive statistics of the data were calculated in the first phase of the statistical analysis. For continuous variables means ± SD, medians and interquartile ranges (IQR) were calculated, while for categorical frequencies, percentages were presented. Demographics, clinical characteristics, and laboratory findings between neonates with Gram-negative vs. Gram-positive pathogens were compared using the non-parametric Wilcoxon rank-sum test or the chi-square test, depending on whether variables were continuous or categorical. To evaluate whether the type of pathogen (Gram-negative vs. Gram-positive) was independently associated with altered haemostatic dynamics, a multivariable linear regression analysis was performed with ROTEM parameters as the dependent variables, and gram type, gender, birth weight, and age of gestation as the independent variables. The Stata 15.0 software (Stata Corp., College Station, TX, USA) was used for the statistical analysis, while statistical significance was set at *p* value < 0.05.

## 5. Conclusions

Sepsis is a complex condition with multiple aetiologies and pathophysiological pathways. The recognition of different host responses according to the type of infectious agent is of critical importance for the choice of the optimal therapeutic strategy. This study has revealed crucial differences in the haemostatic profiles of neonates with septicaemia depending on the type of bacteria. ROTEM showed hypercoagulability in neonates with Gram-positive sepsis, whereas neonates with Gram-negative sepsis had a hypo-coagulant profile and increased incidence of haemorrhagic episodes. These findings highlight the potential for targeted therapeutic strategies based on the causative bacterial pathogen to optimise outcomes in neonatal sepsis. Additionally, this study emphasises the importance of ROTEM for the evaluation of risks of hyper- or hypocoagulability in critical situations such as sepsis. More studies centred around the use of ROTEM in the characterisation of neonatal sepsis are needed to confirm or expand on our findings, and to assess how these can be translated into clinical practice and the improvement of therapeutic strategies for this devastating condition.

## Figures and Tables

**Table 1:** Characteristics of the study population.

	Septic Neonates Due to Gram-Negative Pathogens (Group A = 95)	Septic Neonates Due to Gram-Positive Pathogens (Group B = 33)	*p*-Value
Gestational age (weeks)	32.8 ± 5.2, 32.0 (28.0–38.0)	32.6 ± 3.9, 32.0 (30.0–35.0)	0.91
Gender (males %)	60 (63.1)	21 (63.6)	0.92
Birth weight (g)	1986.6 ± 1104.4, 1480 (1010–2900)	1887.5 ± 967.2, 1490 (1010–2580)	0.97
Cases of death	16 (16.8)	1 (3.0)	**0.044**
Bronchopulmonary dysplasia (BPD)	40 (42.1)	15 (45.4)	0.50
Mild	9 (9.4)	5 (15.1)	
Moderate	2 (2.1)	2 (6.0)	
Severe	29 (30.5)	8 (24.2)	
Retinopathy of prematurity (ROP)	37 (38.9)	13 (39.3)	0.55
Without treatment	26 (27.3)	7 (21.2)	
With treatment	11 (11.5)	6 (18.1)	
Patent ductus arteriosus (PDA)	19 (20.0)	5 (15.1)	0.89
Without treatment	4 (4.2)	1 (3.0)	
Medical treatment	15 (15.7)	4 (12.1)	
Respiratory distress syndrome (RDS)	76 (80.0)	21 (63.6)	0.059
Intraventricular haemorrhage (IVH)	42 (44.2)	18 (51.4)	0.30
Neonatal bleeding assessment tool (NeoBAT)			
No haemorrhage	38 (40)	26 (78.7)	**0.003**
Mild haemorrhage	18 (18.9)	4 (12.1)	
Moderate haemorrhage	15 (15.7)	1 (3.0)	
Major haemorrhage	16 (16.8)	1 (3.0)	
Severe haemorrhage	8 (8.4)	1 (3.0)	
Intrauterine growth retardation (IUGR)	9 (9.4)	3 (8.5)	0.94
Acute kidney injury (AKI)	33 (34.7)	5 (15.1)	**0.034**

**Table 2:** Laboratory data and clinical scores of the study cohort.

Variables	Septic Neonates Due to Gram-Negative Pathogens (Group A = 95)	Septic Neonates Due to Gram-Positive Pathogens (Group B = 33)	*p*-Value
WBC (count × 10^3^/mL)	15.1 ± 9.9, 13.4 (6.8–20.0)	12.1 ± 9.7, 9.6 (7.2–13.3)	**0.037**
Neutrophils (%)	64.1 ± 15.3, 64.0 (54.5–75.3)	55.2 ± 19.7, 53.0 (43.0–70.0)	**0.016**
PLTs (count × 10^3^/mL)	105.8 ± 107.2, 60.0 (25.0–142.0)	178.9 ± 143.0, 169.0 (66.0–280.0)	**0.005**
CRP (mg/L)	72.5 ± 61.4, 53.9 (28.4–108.0)	40.7 ± 40.4, 30.5 (11.0–54.0)	**0.005**
TOLLNER score	11.3 ± 4.3, 11.5 (9.0–14.5)	7.3 ± 4.6, 7.0 (5.0–11.0)	**<0.001**
nSOFA score	5.3 ± 3.7, 5.0 (3.0–8.0)	2.8 ± 3.2, 2.0 (0.0–4.0)	**0.001**
**Modified NEOMOD score**	**7.4 ± 3.2,** **8.0 (5.0–10.0)**	**5.3 ± 2.7,** **5.0 (4.0–7.0)**	**0.002**

Abbreviations: WBC, white blood cells; PLTs, platelets; CRP, C-reactive protein; NEOMOD, neonatal multiple organ dysfunction; nSOFA, neonatal sequential organ failure assessment.

**Table 3:** ROΤΕΜ parameters among the study groups.

Variables	Septic Neonates Due to Gram-negative Pathogens (Group A = 95)	Septic Neonates Due to Gram-positive Pathogens (Group B = 33)	*p*-Value
EXTEM CT (s)	77.1 ± 87.2, 58.0 (51.0–74.0)	53.3 ± 9.4, 51.0 (48.0–60.0)	**0.008**
EXTEM CFT (s)	283.7 ± 375.3, 151.0 (90.0–347.5)	146.6 ± 207.5, 87.0 (67.0–129.0)	**0.001**
EXTEM A10 (mm)	39.4 ± 16.3, 40.0 (26.0–53.0)	49.3 ± 13.5, 51.5 (41.0–58.5)	**0.001**
EXTEM MCF (mm)	47.8 ± 16.0, 49.0 (36.0–59.0)	58.8 ± 13.3, 61.0 (53.0–66.0)	**<0.001**
EXTEM Alpha angle (°)	67.4 ± 15.0, 72.5 (61.0–78.0)	75.6 ± 5.5, 76.0 (73.0–79.0)	**0.004**
EXTEM LI60 (%)	96.2 ± 4.2, 98.0 (94.0–100.0)	94.2 ± 4.0, 93.0 (91.0–98.0)	**0.011**
INTEM CT (s)	293.0 ± 560.1, 221.0 (177.5–278.0)	196.9 ± 59.8, 206.5 (166.0–226.0)	0.11
INTEM CFT (s)	326.3 ± 495.8, 156.0 (90.0–367.0)	156.7 ± 281.9, 89.5 (58.0–126.5)	**<0.001**
INTEM A10 (mm)	38.6 ± 15.9, 39.0 (24.0–51.0)	48.4 ± 13.1, 47.0 (42.0–58.0)	**0.003**
INTEM MCF (mm)	47.3 ± 14.6, 49.0 (35.0–58.0)	55.2 ± 10.2, 56.5 (48.5–61.5)	**0.008**
INTEM Alpha angle (°)	63.5 ± 13.9, 66.0 (57.0–74.0)	72.7 ± 7.1, 73.5 (69.5–78.0)	**<0.001**
INTEM LI60 (%)	96.6 ± 3.8, 98.0 (95.0–100.0)	95.0 ± 4.4, 95.0 (91.0–99.0)	0.12
FIBTEM CT (s)	179.7 ± 789.9, 55.0 (46.0–67.0)	49.6 ± 11.8, 50.5 (45.0–55.0)	**0.042**
FIBTEM A10 (mm)	17.2 ± 7.6, 17.0 (12.0–22.0)	22.5 ± 9.7, 20.0 (18.0–27.0)	**0.004**
FIBTEM MCF (mm)	22.2 ± 11.4, 20.0 (14.0–28.0)	25.1 ± 10.6, 23.5 (19.0–29.0)	0.16
FIBTEM Alpha angle (°)	74.2 ± 11.0, 77.0 (72.0–80.0)	78.0 ± 5.8, 78.5 (76.0–82.0)	**0.038**
FIBTEM LI60 (%)	99.4 ± 2.4, 100.0 (100.0–100.0)	98.8 ± 3.8, 100.0 (100.0–100.0)	0.40

Abbreviations: CT, clotting time; CFT, clot formation time; A10, clot amplitude at 10 min; MCF, maximum clot firmness; LI60, lysis index at 60 min.

**Table 4:** Results of multivariable regression analysis for ROTEM parameters as dependent variables, with gestational age, birth weight, sex, and type of pathogen (Gram-positive vs. Gram-negative) as independent variables.

ROTEM Parameters	Gram-Positive Pathogen		
	Coefficient	95% CI	*p*-Value
EXTEM CT (s)	−24.5	−54.7–5.6	0.11
EXTEM CFT (s)	−121.7	−252.6–9.0	0.06
EXTEM A10 (mm)	8.9	2.8–15.0	**0.004**
EXTEM MCF (mm)	10.4	4.3–16.6	**0.001**
EXTEM alpha angle (°)	8.0	2.7–13.2	**0.003**
EXTEM LI60 (%)	−1.8	−3.6–0.001	0.050
INTEM CT (s)	−97.7	−309.2–113.7	0.36
INTEM CFT (s)	−153.7	−352.4–44.8	0.12
INTEM A10 (mm)	8.7	2.1–15.2	**0.009**
INTEM MCF (mm)	7.1	1.2–13.0	**0.017**
INTEM alpha angle (°)	8.5	3.1–13.9	**0.002**
INTEM LI60 (%)	−1.4	−3.3–0.3	0.11
FIBTEM CT (s)	−110.7	−398.3–176.7	0.44
FIBTEM A10 (mm)	5.0	1.4–8.5	**0.006**
FIBTEM MCF (mm)	2.3	−2.4–7.0	0.33
FIBTEM alpha angle (°)	3.9	−0.7–8.1	0.10
FIBTEM LI60 (%)	−0.6	−1.9–0.7	0.37

Abbreviations: CI, confidence interval; CT, clotting time; CFT, clot formation time; A10, clot amplitude at 10 min; MCF, maximum clot firmness; LI60, lysis index at 60 min.

## Data Availability

The original contributions presented in the study are included in the article, further inquiries can be directed to the corresponding author.

## References

[1] Singer M., Deutschman C.S., Seymour C.W., Shankar-Hari M., Annane D., Bauer M., Bellomo R., Bernard G.R., Chiche J.D., Coopersmith C.M. (2016). The Third International Consensus Definitions for Sepsis and Septic Shock (Sepsis-3). JAMA.

[2] Fleischmann-Struzek C., Goldfarb D.M., Schlattmann P., Schlapbach L.J., Reinhart K., Kissoon N. (2018). The global burden of paediatric and neonatal sepsis: A systematic review. Lancet Respir. Med..

[3] Pappas P.G., Lionakis M.S., Arendrup M.C., Ostrosky-Zeichner L., Kullberg B.J. (2018). Invasive candidiasis. Nat. Rev. Dis. Primers.

[4] (2018). Global, regional, and national incidence, prevalence, and years lived with disability for 354 diseases and injuries for 195 countries and territories, 1990-2017: A systematic analysis for the Global Burden of Disease Study 2017. Lancet.

[5] Kopanou Taliaka P., Tsantes A.G., Konstantinidi A., Houhoula D., Tsante K.A., Vaiopoulos A.G., Piovani D., Nikolopoulos G.K., Bonovas S., Iacovidou N. (2023). Risk Factors, Diagnosis, and Treatment of Neonatal Fungal Liver Abscess: A Systematic Review of the Literature. Life.

[6] Nwankwor O.C., McKelvie B., Frizzola M., Hunter K., Kabara H.S., Oduwole A., Oguonu T., Kissoon N. (2019). A National Survey of Resources to Address Sepsis in Children in Tertiary Care Centers in Nigeria. Front. Pediatr..

[7] Ranjeva S.L., Warf B.C., Schiff S.J. (2018). Economic burden of neonatal sepsis in sub-Saharan Africa. BMJ Glob. Health.

[8] Bruns N., Dohna-Schwake C. (2022). Antibiotics in critically ill children-a narrative review on different aspects of a rational approach. Pediatr. Res..

[9] Molloy E.J., Bearer C.F. (2022). Paediatric and neonatal sepsis and inflammation. Pediatr. Res..

[10] Hornik C.P., Fort P., Clark R.H., Watt K., Benjamin D.K., Smith P.B., Manzoni P., Jacqz-Aigrain E., Kaguelidou F., Cohen-Wolkowiez M. (2012). Early and late onset sepsis in very-low-birth-weight infants from a large group of neonatal intensive care units. Early Hum. Dev..

[11] Cernada M., Pinilla-González A., Kuligowski J., Morales J.M., Lorente-Pozo S., Piñeiro-Ramos J.D., Parra-Llorca A., Lara-Cantón I., Vento M., Serna E. (2022). Transcriptome profiles discriminate between Gram-positive and Gram-negative sepsis in preterm neonates. Pediatr. Res..

[12] Mahich S., Angurana S.K., Sundaram V., Gautam V. (2022). Epidemiology, microbiological profile, and outcome of culture positive sepsis among outborn neonates at a tertiary hospital in Northern India. J. Matern.-Fetal Neonatal Med. Off. J. Eur. Assoc. Perinat. Med. Fed. Asia Ocean. Perinat. Soc. Int. Soc. Perinat. Obs..

[13] Abe R., Oda S., Sadahiro T., Nakamura M., Hirayama Y., Tateishi Y., Shinozaki K., Hirasawa H. (2010). Gram-negative bacteremia induces greater magnitude of inflammatory response than Gram-positive bacteremia. Crit. Care.

[14] Lutsar I., Chazallon C., Carducci F.I., Trafojer U., Abdelkader B., de Cabre V.M., Esposito S., Giaquinto C., Heath P.T., Ilmoja M.L. (2014). Current management of late onset neonatal bacterial sepsis in five European countries. Eur. J. Pediatr..

[15] Bethou A., Bhat B.V. (2022). Neonatal Sepsis-Newer Insights. Indian J. Pediatr..

[16] Hornik C.P., Becker K.C., Benjamin D.K., Li J., Clark R.H., Cohen-Wolkowiez M., Brian Smith P. (2012). Use of the complete blood cell count in late-onset neonatal sepsis. Pediatr. Infect. Dis. J..

[17] Garland S.M., Bowman E.D. (2003). Reappraisal of C-reactive protein as a screening tool for neonatal sepsis. Pathology.

[18] Aloisio E., Dolci A., Panteghini M. (2019). Procalcitonin: Between evidence and critical issues. Clin. Chim. Acta Int. J. Clin. Chem..

[19] Gopal N., Chauhan N., Jain U., Dass S.K., Sharma H.S., Chandra R. (2023). Advancement in biomarker based effective diagnosis of neonatal sepsis. Artif. Cells Nanomed. Biotechnol..

[20] Kamel M.M., Abd-Ullah H.F., El Sayed M.A., Abdel Aziz R.A. (2021). Presepsin as an early predictor of neonatal sepsis. Int. J. Pediatr..

[21] El-Masry H.M., Hassan A.-E.A., Amin H.H., Abd Elmoaty M.A. (2021). Evaluation of serum presepsin concentrations as a biomarker of sepsis in high-risk neonates. Al-Azhar Assiut Med. J..

[22] Poggi C., Bianconi T., Gozzini E., Generoso M., Dani C. (2015). Presepsin for the detection of late-onset sepsis in preterm newborns. Pediatrics.

[23] Montaldo P., Rosso R., Santantonio A., Chello G., Giliberti P. (2017). Presepsin for the detection of early-onset sepsis in preterm newborns. Pediatr. Res..

[24] Kumar N., Dayal R., Singh P., Pathak S., Pooniya V., Goyal A., Kamal R., Mohanty K.K. (2019). A Comparative Evaluation of Presepsin with Procalcitonin and CRP in Diagnosing Neonatal Sepsis. Indian J. Pediatr..

[25] Zhang J., Chen L., Yang Y., Liu X., Yuan Y., Song S.R., Zhao Y., Mao J. (2023). Clinical and laboratory findings to differentiate late-onset sepsis caused by Gram-negative vs Gram-positive bacteria among perterm neonates: A retrospective cohort study. Int. Immunopharmacol..

[26] Zeerleder S., Hack C.E., Wuillemin W.A. (2005). Disseminated intravascular coagulation in sepsis. Chest.

[27] Walsh T.S., Stanworth S.J., Prescott R.J., Lee R.J., Watson D.M., Wyncoll D. (2010). Prevalence, management, and outcomes of critically ill patients with prothrombin time prolongation in United Kingdom intensive care units. Crit. Care Med..

[28] Saito S., Uchino S., Hayakawa M., Yamakawa K., Kudo D., Iizuka Y., Sanui M., Takimoto K., Mayumi T., Sasabuchi Y. (2019). Epidemiology of disseminated intravascular coagulation in sepsis and validation of scoring systems. J. Crit. Care.

[29] ten Cate H. (2000). Pathophysiology of disseminated intravascular coagulation in sepsis. Crit. Care Med..

[30] Renckens R., Roelofs J.J., Stegenga M.E., Florquin S., Levi M., Carmeliet P., Van’t Veer C., van der Poll T. (2008). Transgenic tissue-type plasminogen activator expression improves host defense during Klebsiella pneumonia. J. Thromb. Haemost. JTH.

[31] Lichota A., Gwozdzinski K., Szewczyk E.M. (2020). Microbial Modulation of Coagulation Disorders in Venous Thromboembolism. J. Inflamm. Res..

[32] Carrol E.D., Ranjit S., Menon K., Bennett T.D., Sanchez-Pinto L.N., Zimmerman J.J., Souza D.C., Sorce L.R., Randolph A.G., Ishimine P. (2023). Operationalizing Appropriate Sepsis Definitions in Children Worldwide: Considerations for the Pediatric Sepsis Definition Taskforce. Pediatr. Crit. Care Med..

[33] Horn D.L., Morrison D.C., Opal S.M., Silverstein R., Visvanathan K., Zabriskie J.B. (2000). What are the microbial components implicated in the pathogenesis of sepsis? Report on a symposium. Clin. Infect. Dis. Off. Publ. Infect. Dis. Soc. Am..

[34] Sriskandan S., Cohen J. (1999). Gram-positive sepsis. Mechanisms and differences from gram-negative sepsis. Infect. Dis. Clin. N. Am..

[35] León C., Rodrigo M.J., Tomasa A., Gallart M.T., Latorre F.J., Rius J., Brugués J. (1982). Complement activation in septic shock due to gram-negative and gram-positive bacteria. Crit. Care Med..

[36] Tang A., Shi Y., Dong Q., Wang S., Ge Y., Wang C., Gong Z., Zhang W., Chen W. (2023). Prognostic differences in sepsis caused by gram-negative bacteria and gram-positive bacteria: A systematic review and meta-analysis. Crit. Care.

[37] Angus D.C., Wax R.S. (2001). Epidemiology of sepsis: An update. Crit. Care Med..

[38] Ahmed A.J., Kruse J.A., Haupt M.T., Chandrasekar P.H., Carlson R.W. (1991). Hemodynamic responses to gram-positive versus gram-negative sepsis in critically ill patients with and without circulatory shock. Crit. Care Med..

[39] Natanson C., Danner R.L., Elin R.J., Hosseini J.M., Peart K.W., Banks S.M., MacVittie T., Walker R., Parrillo J. (1989). Role of endotoxemia in cardiovascular dysfunction and mortality. Escherichia coli and Staphylococcus aureus challenges in a canine model of human septic shock. J. Clin. Investig..

[40] Fisher C.J., Agosti J.M., Opal S.M., Lowry S.F., Balk R.A., Sadoff J.C., Abraham E., Schein R.M., Benjamin E. (1996). Treatment of septic shock with the tumor necrosis factor receptor: Fc fusion protein. N. Engl. J. Med..

[41] Zeni F., Freeman B., Natanson C. (1997). Anti-inflammatory therapies to treat sepsis and septic shock: A reassessment. Crit. Care Med..

[42] Opal S.M., Cohen J. (1999). Clinical gram-positive sepsis: Does it fundamentally differ from gram-negative bacterial sepsis?. Crit. Care Med..

[43] Opal S.M., Garber G.E., LaRosa S.P., Maki D.G., Freebairn R.C., Kinasewitz G.T., Dhainaut J.-F., Yan S.B., Williams M.D., Graham D.E. (2003). Systemic Host Responses in Severe Sepsis Analyzed by Causative Microorganism and Treatment Effects of Drotrecogin Alfa (Activated). Clin. Infect. Dis..

[44] Sokou R., Foudoulaki-Paparizos L., Lytras T., Konstantinidi A., Theodoraki M., Lambadaridis I., Gounaris A., Valsami S., Politou M., Gialeraki A. (2017). Reference ranges of thromboelastometry in healthy full-term and pre-term neonates. Clin. Chem. Lab. Med..

[45] Liu Q., Xu C., Chen X., Wang J., Ke Z., Hu H. (2019). Establishing a reference range for thromboelastograph parameters in the neonatal period. Int. J. Lab. Hematol..

[46] Theodoraki M., Sokou R., Valsami S., Iliodromiti Z., Pouliakis A., Parastatidou S., Karavana G., Ioakeimidis G., Georgiadou P., Iacovidou N. (2020). Reference Values of Thrombolastometry Parameters in Healthy Term Neonates. Children.

[47] Ravn H.B., Andreasen J.B., Hvas A.M. (2017). Does whole blood coagulation analysis reflect developmental haemostasis?. Blood Coagul. Fibrinolysis Int. J. Haemost. Thromb..

[48] Sewell E.K., Forman K.R., Wong E.C., Gallagher M., Luban N.L., Massaro A.N. (2017). Thromboelastography in term neonates: An alternative approach to evaluating coagulopathy. Arch. Dis. Child. Fetal Neonatal Ed..

[49] Motta M., Guaragni B., Pezzotti E., Rodriguez-Perez C., Chirico G. (2017). Reference intervals of citrated-native whole blood thromboelastography in premature neonates. Early Hum. Dev..

[50] Oswald E., Stalzer B., Heitz E., Weiss M., Schmugge M., Strasak A., Innerhofer P., Haas T. (2010). Thromboelastometry (ROTEM) in children: Age-related reference ranges and correlations with standard coagulation tests. Br. J. Anaesth..

[51] Soerensen K., Olsen H., Skovgaard K., Wiinberg B., Nielsen O., Leifsson P., Jensen H., Kristensen A., Iburg T. (2013). Disseminated intravascular coagulation in a novel porcine model of severe Staphylococcus aureus sepsis fulfills human clinical criteria. J. Comp. Pathol..

[52] Krogh A.K.H., Brunse A., Thymann T., Bochsen L., Kristensen A.T. (2019). Staphylococcus epidermidis sepsis induces hypercoagulability in preterm pigs. Res. Vet. Sci..

[53] Görlinger K., Dirkmann D., Albrecht A. (2016). Rotational thromboelastometry (ROTEM^®^). Trauma Induced Coagulopathy.

[54] Raspé C., Besch M., Charitos E.I., Flöther L., Bucher M., Rückert F., Treede H. (2018). Rotational Thromboelastometry for Assessing Bleeding Complications and Factor XIII Deficiency in Cardiac Surgery Patients. Clin. Appl. Thromb./Hemost. Off. J. Int. Acad. Clin. Appl. Thromb./Hemost..

[55] Sola M.C., Calhoun D.A., Hutson A.D., Christensen R.D. (1999). Plasma thrombopoietin concentrations in thrombocytopenic and non-thrombocytopenic patients in a neonatal intensive care unit. Br. J. Haematol..

[56] Colarizi P., Fiorucci P., Caradonna A., Ficuccilli F., Mancuso M., Papoff P. (1999). Circulating thrombopoietin levels in neonates with infection. Acta Paediatr..

[57] Guida J.D., Kunig A.M., Leef K.H., McKenzie S.E., Paul D.A. (2003). Platelet count and sepsis in very low birth weight neonates: Is there an organism-specific response?. Pediatrics.

[58] Manzoni P., Mostert M., Galletto P., Gastaldo L., Gallo E., Agriesti G., Farina D. (2009). Is thrombocytopenia suggestive of organism-specific response in neonatal sepsis?. Pediatr. Int. Off. J. Jpn. Pediatr. Soc..

[59] Khashu M., Osiovich H., Henry D., Khotani A.A., Solimano A., Speert D.P. (2006). Persistent bacteremia and severe thrombocytopenia caused by coagulase-negative Staphylococcus in a neonatal intensive care unit. Pediatrics.

[60] Cordero L., Rau R., Taylor D., Ayers L.W. (2004). Enteric gram-negative bacilli bloodstream infections: 17 years’ experience in a neonatal intensive care unit. Am. J. Infect. Control.

[61] Eissa D.S., El-Farrash R.A. (2013). New insights into thrombopoiesis in neonatal sepsis. Platelets.

[62] Scheifele D.W., Olsen E.M., Pendray M.R. (1985). Endotoxinemia and thrombocytopenia during neonatal necrotizing enterocolitis. Am. J. Clin. Pathol..

[63] Rowe M.I., Buckner D.M., Newmark S. (1975). The early diagnosis of gram negative septicemia in the pediatric surgical patient. Ann. Surg..

[64] Sheu J.-R., Hung W.-C., Wu C.-H., Ma M.-C., Kan Y.-C., Lin C.-H., Lin M.-S., Luk H.-N., Yen M.-H. (1999). Reduction in Lipopolysaccharide-Induced Thrombocytopenia by Triflavin in a Rat Model of Septicemia. Circulation.

[65] Ginsberg M.H., Henson P.M. (1978). Enhancement of platelet response to immune complexes and IgG aggregates by lipid A-rich bacterial lipopolysaccharides. J. Exp. Med..

[66] Ginsberg M.H., Morrison D.C. (1978). The selective binding of aggregated IgG to lipid A-rich bacterial lipopolysaccharides. J. Immunol..

[67] Im S.Y., Choi J.H., Ko H.M., Han S.J., Chun S.B., Lee H.K., Ha T.Y. (1997). A protective role of platelet-activating factor in murine candidiasis. Infect. Immun..

[68] Hamzeh-Cognasse H., Damien P., Chabert A., Pozzetto B., Cognasse F., Garraud O. (2015). Platelets and infections—Complex interactions with bacteria. Front. Immunol..

[69] Arman M., Krauel K. (2015). Human platelet IgG Fc receptor FcγRIIA in immunity and thrombosis. J. Thromb. Haemost. JTH.

[70] Thammavongsa V., Kim H.K., Missiakas D., Schneewind O. (2015). Staphylococcal manipulation of host immune responses. Nat. Rev. Microbiol..

[71] Li X., Wang S., Ma J., Bai S.G., Fu S.Z. (2024). Predictive value of thrombocytopenia for bloodstream infection in patients with sepsis and septic shock. World J. Crit. Care Med..

[72] Ezzeroug Ezzraimi A., Hannachi N., Mariotti A., Rolain J.-M., Camoin-Jau L. (2022). Platelets and Escherichia coli: A Complex Interaction. Biomedicines.

[73] Fitzgerald J.R., Foster T.J., Cox D. (2006). The interaction of bacterial pathogens with platelets. Nat. Rev. Microbiol..

[74] Rotstein O. (1992). Role of fibrin deposition in the pathogenesis of intraabdominal infection. Eur. J. Clin. Microbiol. Infect. Dis..

[75] Sun H., Wang X., Degen J.L., Ginsburg D. (2009). Reduced thrombin generation increases host susceptibility to group A streptococcal infection. Blood.

[76] Wang Z., Wilhelmsson C., Hyrsl P., Loof T.G., Dobes P., Klupp M., Loseva O., Mörgelin M., Iklé J., Cripps R.M. (2010). Pathogen entrapment by transglutaminase—A conserved early innate immune mechanism. PLoS Pathog..

[77] Loof T.G., Mörgelin M., Johansson L., Oehmcke S., Olin A.I., Dickneite G., Norrby-Teglund A., Theopold U., Herwald H. (2011). Coagulation, an ancestral serine protease cascade, exerts a novel function in early immune defense. Blood.

[78] Blombäck B., Procyk R., Adamson L., Hessel B. (1985). FXIII induced gelation of human fibrinogen—An alternative thiol enhanced, thrombin independent pathway. Thromb. Res..

[79] Okada M., Blombäck B., Chang M.D., Horowitz B. (1985). Fibronectin and fibrin gel structure. J. Biol. Chem..

[80] Chow T.W., McIntire L.V., Peterson D.M. (1983). Importance of plasma fibronectin in determining PFP and PRP clot mechanical properties. Thromb. Res..

[81] Matsuka Y.V., Anderson E.T., Milner-Fish T., Ooi P., Baker S. (2003). Staphylococcus aureus fibronectin-binding protein serves as a substrate for coagulation factor XIIIa: Evidence for factor XIIIa-catalyzed covalent cross-linking to fibronectin and fibrin. Biochemistry.

[82] Thomer L., Schneewind O., Missiakas D. (2013). Multiple ligands of von Willebrand factor-binding protein (vWbp) promote Staphylococcus aureus clot formation in human plasma. J. Biol. Chem..

[83] Wynn J.L., Wong H.R. (2010). Pathophysiology and treatment of septic shock in neonates. Clin. Perinatol..

[84] van der Poll T., Levi M. (2012). Crosstalk between inflammation and coagulation: The lessons of sepsis. Curr. Vasc. Pharmacol..

[85] Degen J.L., Bugge T.H., Goguen J.D. (2007). Fibrin and fibrinolysis in infection and host defense. J. Thromb. Haemost. JTH.

[86] Schmitt F.C.F., Manolov V., Morgenstern J., Fleming T., Heitmeier S., Uhle F., Al-Saeedi M., Hackert T., Bruckner T., Schöchl H. (2019). Acute fibrinolysis shutdown occurs early in septic shock and is associated with increased morbidity and mortality: Results of an observational pilot study. Ann. Intensive Care.

[87] Gomez-Builes J.C., Acuna S.A., Nascimento B., Madotto F., Rizoli S.B. (2018). Harmful or Physiologic: Diagnosing Fibrinolysis Shutdown in a Trauma Cohort with Rotational Thromboelastometry. Anesth. Analg..

[88] Lampridou M., Sokou R., Tsantes A.G., Theodoraki M., Konstantinidi A., Ioakeimidis G., Bonovas S., Politou M., Valsami S., Iliodromiti Z. (2020). ROTEM diagnostic capacity for measuring fibrinolysis in neonatal sepsis. Thromb. Res..

[89] Sokou R., Giallouros G., Konstantinidi A., Pantavou K., Nikolopoulos G., Bonovas S., Lytras T., Kyriakou E., Lambadaridis I., Gounaris A. (2018). Thromboelastometry for diagnosis of neonatal sepsis-associated coagulopathy: An observational study. Eur. J. Pediatr..

[90] Sokou R., Ioakeimidis G., Piovani D., Parastatidou S., Konstantinidi A., Tsantes A.G., Lampridou M., Houhoula D., Iacovidou N., Kokoris S. (2022). Development and validation of a sepsis diagnostic scoring model for neonates with suspected sepsis. Front. Pediatr..

[91] Sokou R., Tsantes A.G., Lampridou M., Tsante K.A., Houhoula D., Piovani D., Bonovas S., Boutsikou T., Iliodromiti Z., Iacovidou N. (2024). Thromboelastometry and prediction of in-hospital mortality in neonates with sepsis. Int. J. Lab. Hematol..

[92] Sokou R., Georgiadou P., Tsantes A.G., Parastatidou S., Konstantinidi A., Ioakeimidis G., Makrogianni A., Theodoraki M., Kokoris S., Iacovidou N. (2023). The Utility of NATEM Assay in Predicting Bleeding Risk in Critically Ill Neonates. Semin. Thromb. Hemost..

[93] Sokou R., Tsantes A.G., Konstantinidi A., Ioakeimidis G., Lampridou M., Parastatidou S., Theodoraki M., Piovani D., Iliodromiti Z., Boutsikou T. (2021). Rotational Thromboelastometry in Neonates Admitted to a Neonatal Intensive Care Unit: A Large Cross-sectional Study. Semin. Thromb. Hemost..

[94] Sokou R., Tritzali M., Piovani D., Konstantinidi A., Tsantes A.G., Ioakeimidis G., Lampridou M., Parastatidou S., Iacovidou N., Kokoris S. (2021). Comparative Performance of Four Established Neonatal Disease Scoring Systems in Predicting In-Hospital Mortality and the Potential Role of Thromboelastometry. Diagnostics.

[95] Tsantes A.G., Konstantinidi A., Parastatidou S., Ioakeimidis G., Tsante K.A., Mantzios P.G., Kriebardis A.G., Gialeraki A., Houhoula D., Iacovidou N. (2022). Assessment of agreement between EXTEM and NATEM thromboelastometry measurement assays in critically ill neonates. Eur. J. Haematol..

[96] Adamzik M., Langemeier T., Frey U.H., Gorlinger K., Saner F., Eggebrecht H., Peters J., Hartmann M. (2011). Comparison of thrombelastometry with simplified acute physiology score II and sequential organ failure assessment scores for the prediction of 30-day survival: A cohort study. Shock.

[97] Ostrowski S.R., Windelov N.A., Ibsen M., Haase N., Perner A., Johansson P.I. (2013). Consecutive thrombelastography clot strength profiles in patients with severe sepsis and their association with 28-day mortality: A prospective study. J. Crit. Care.

[98] Munford R.S. (2006). SEVERE SEPSIS AND SEPTIC SHOCK: The Role of Gram-Negative Bacteremia. Annu. Rev. Pathol. Mech. Dis..

[99] Alexandraki I., Palacio C. (2010). Gram-negative versus Gram-positive bacteremia: What is more alarmin(g)?. Crit. Care.

[100] Webb S.A., Kahler C.M. (2008). Bench-to-bedside review: Bacterial virulence and subversion of host defences. Crit. Care.

[101] Finlay B.B., McFadden G. (2006). Anti-immunology: Evasion of the host immune system by bacterial and viral pathogens. Cell.

[102] Tsantes A.G., Parastatidou S., Tsantes E.A., Bonova E., Tsante K.A., Mantzios P.G., Vaiopoulos A.G., Tsalas S., Konstantinidi A., Houhoula D. (2023). Sepsis-Induced Coagulopathy: An Update on Pathophysiology, Biomarkers, and Current Guidelines. Life.

[103] Toh C.H., Dennis M. (2003). Disseminated intravascular coagulation: Old disease, new hope. BMJ.

[104] Sokou R., Parastatidou S., Konstantinidi A., Tsantes A.G., Iacovidou N., Piovani D., Bonovas S., Tsantes A.E. (2024). Contemporary tools for evaluation of hemostasis in neonates. Where are we and where are we headed?. Blood Rev..

[105] Sokou R., Parastatidou S., Konstantinidi A., Tsantes A.G., Iacovidou N., Piovani D., Bonovas S., Tsantes A.E. (2024). Bleeding Scoring Systems in Neonates: A Systematic Review. Semin. Thromb. Hemost..

[106] Sokou R., Parastatidou S., Konstantinidi A., Tsantes A.G., Iacovidou N., Doxani C., Piovani D., Bonovas S., Stefanidis I., Zintzaras E. (2022). Fresh frozen plasma transfusion in the neonatal population: A systematic review. Blood Rev..

[107] Toh C.H., Samis J., Downey C., Walker J., Becker L., Brufatto N., Tejidor L., Jones G., Houdijk W., Giles A. (2002). Biphasic transmittance waveform in the APTT coagulation assay is due to the formation of a Ca(++)-dependent complex of C-reactive protein with very-low-density lipoprotein and is a novel marker of impending disseminated intravascular coagulation. Blood.

[108] Rivard G.E., Brummel-Ziedins K.E., Mann K.G., Fan L., Hofer A., Cohen E. (2005). Evaluation of the profile of thrombin generation during the process of whole blood clotting as assessed by thrombelastography. J. Thromb. Haemost..

[109] Guenter L., Mayr V., Fries D., Innerhofer P., Jochberger S., Hasibeder W., Dunser M. (2008). Uncovering Hypercoagulability in Sepsis Using ROTEM^®^ Thromboelastometry: A Case Series. Open Crit. Care Med. J..

[110] Ramsay M.A., Randall H.B., Burton E.C. (2004). Intravascular thrombosis and thromboembolism during liver transplantation: Antifibrinolytic therapy implicated?. Liver Transplant. Off. Publ. Am. Assoc. Study Liver Dis. Int. Liver Transplant. Soc..

[111] Leligdowicz A., Matthay M.A. (2019). Heterogeneity in sepsis: New biological evidence with clinical applications. Crit. Care.

[112] Russell C.D., Baillie J.K. (2017). Treatable traits and therapeutic targets: Goals for systems biology in infectious disease. Curr. Opin. Syst. Biol..

[113] McGovern M., Giannoni E., Kuester H., Turner M.A., van den Hoogen A., Bliss J.M., Koenig J.M., Keij F.M., Mazela J., Finnegan R. (2020). Challenges in developing a consensus definition of neonatal sepsis. Pediatr. Res..

[114] Levy M.M., Fink M.P., Marshall J.C., Abraham E., Angus D., Cook D., Cohen J., Opal S.M., Vincent J.L., Ramsay G. (2003). 2001 SCCM/ESICM/ACCP/ATS/SIS International Sepsis Definitions Conference. Crit. Care Med..

[115] Wynn J.L., Polin R.A. (2020). A neonatal sequential organ failure assessment score predicts mortality to late-onset sepsis in preterm very low birth weight infants. Pediatr. Res..

[116] Fleiss N., Coggins S.A., Lewis A.N., Zeigler A., Cooksey K.E., Walker L.A., Husain A.N., de Jong B.S., Wallman-Stokes A., Alrifai M.W. (2021). Evaluation of the Neonatal Sequential Organ Failure Assessment and Mortality Risk in Preterm Infants with Late-Onset Infection. JAMA Netw. Open.

[117] Töllner U. (1982). Early diagnosis of septicemia in the newborn. Clinical studies and sepsis score. Eur. J. Pediatr..

[118] Çetinkaya M., Köksal N., Özkan H. (2012). A New Scoring System For Evaluation of Multiple Organ Dysfunction Syndrome in Premature Infants. Am. J. Crit. Care.

[119] Venkatesh V., Curley A., Khan R., Clarke P., Watts T., Josephson C., Muthukumar P., New H., Seeney F., Morris S. (2013). A novel approach to standardised recording of bleeding in a high risk neonatal population. Arch. Dis. Child. Fetal Neonatal Ed..

